# Association between Body Roundness Index and Functional Impairment in Older Adults: Exploring the Potential Mediating Role of Systemic Inflammation Response Index

**DOI:** 10.1016/j.cdnut.2026.107671

**Published:** 2026-03-12

**Authors:** Min Zhou, Cui Mao, Yanying Yang, Huiping Qiu

**Affiliations:** 1 Department of Medicine, Quzhou College of Technology, Quzhou City, China; 2 Department of Nursing, Henan Vocational College of Nursing, Anyang City, China

**Keywords:** body roundness index, functional impairment, physical limitation, systemic inflammation response index, older adults

## Abstract

**Background:**

Functional impairment (FI) represents a major health challenge among older adults.

**Objectives:**

This study aimed to investigate the association between the body roundness index (BRI), a novel indicator of central obesity, and FI, as well as to examine the mediating role of the systemic inflammation response index (SIRI) in this relationship.

**Methods:**

Data were obtained from the NHANES (1999–2018), comprising 15,110 older adults. BRI was calculated using waist circumference and height. FI was defined based on physical limitations, basic activities of daily living, and instrumental activities of daily living. SIRI was derived from peripheral blood cell counts. Weighted multivariable logistic regression, restricted cubic spline (RCS) curves, subgroup analyses, mediation analysis, and sensitivity analyses were performed.

**Results:**

Among the participants, 9734 individuals were identified as having FI. In the fully adjusted model, BRI was significantly positively associated with the presence of FI [odds ratio (OR): 1.70; 95% confidence interval (CI): 1.60, 1.80]. A similar positive association was observed between SIRI and FI (OR: 1.23; 95% CI: 1.16, 1.32). RCS curves revealed a nonlinear dose-response relationship between BRI and FI (*P*-overall < 0.001; *P*-nonlinearity < 0.001) and between SIRI and FI (*P*-overall < 0.001; *P*-nonlinearity = 0.016). Notably, mediation analysis indicated that SIRI partially mediated the association between BRI and FI in older adults (indirect effect = 0.00116; *P* < 0.001; mediated proportion = 2.23%).

**Conclusions:**

These findings may help elucidate the potential mechanisms linking central obesity to FI in older adults, offering insights for targeted prevention of functional disability.

## Introduction

As life expectancy increases and the proportion of older adults continues to grow, this population faces not only psychological challenges, such as social isolation [1], but also a progressive decline or loss of basic functional abilities [2]. In particular, the functional impairment (FI) among older adults has been rising [3], with the absolute number of affected individuals increasing annually [4]. FI is typically assessed based on self-reported difficulty or the need for assistance with daily activities, encompassing basic activities of daily living (BADL) (e.g., bathing and dressing) as well as more complex tasks such as using the telephone, managing medications, or preparing meals [5]. The consequences of FI are profound and include an increased risk of urinary incontinence [6], venous thrombosis [7], nursing home placement [8,9], emergency department visits and hospitalization [10], and even mortality [11]. Therefore, identifying risk factors associated with FI in older adults is of critical public health importance.

The body roundness index (BRI), first introduced by Thomas et al. [12] in 2013, is a novel anthropometric indicator designed to assess body fat and visceral fat content by integrating waist circumference (WC) and height, thereby reflecting the degree of central obesity. Although WC remains a simple and clinically practical measure of central adiposity, it may be systematically influenced by an individual’s height. In contrast, BRI may offer a more refined assessment of visceral fat accumulation and its associated health risks. Compared with traditional anthropometric indices such as BMI and WC, BRI has been shown to more accurately reflect body fat percentage and visceral fat levels [13]. As a result, BRI may hold greater promise for evaluating health status in middle-aged and older adults and has potential value for clinical application. Emerging evidence suggests that BRI is effective in predicting the risk of metabolic syndrome [14] and hypertension [15]. Nevertheless, research examining the association between BRI and FI in older adults remains limited.

Previous studies have demonstrated that individuals with obesity often exhibit a persistent subclinical inflammatory state, which may contribute to the development of chronic inflammation [16]. The systemic inflammation response index (SIRI) is a novel marker of chronic low-grade inflammation, derived from peripheral blood counts of monocytes, neutrophils, and lymphocytes [17]. It is calculated by multiplying the neutrophil count by the monocyte count and then dividing by the lymphocyte count [18]. SIRI reflects the interplay between local immune responses and systemic inflammation, encompassing interactions between innate and adaptive immunity, the balance between proinflammatory and anti-inflammatory processes, and the modulation of inflammation-related immune networks across organs [19]. SIRI has been increasingly recognized as a valuable indicator for assessing health outcomes in older adults, including cardiovascular disease [20], chronic obstructive pulmonary disease (COPD) [21], and various tumors [22]. A persistent inflammatory state may lead to tissue damage, resulting in functional abnormalities and, ultimately, FI [23]. Emerging evidence suggests that systemic inflammation in older adults is associated with an increased risk of FI [24,25]. Moreover, inflammatory markers have been shown to mediate the relationship between BRI and cardiometabolic risk [26]. However, whether SIRI mediates the association between BRI and FI in older adults remains unexplored.

Although traditional anthropometric measures such as BMI and WC remain widely used in both research and clinical settings, the BRI offers a geometrically derived estimate of visceral adiposity that may more accurately capture health risks associated with body shape. Similarly, although well-established inflammatory markers such as C-reactive protein (CRP) and IL-6 are commonly employed in aging research, the SIRI provides a composite, clinically accessible measure derived from routine complete blood counts (CBCs). The present study aims to investigate whether these novel indices contribute unique insights into the pathway linking central obesity to FI. Specifically, we seek to examine the association between BRI and FI in a large, representative sample of older adults and to further determine the mediating role of SIRI in this relationship. Our findings may offer a novel perspective on the interplay between visceral obesity and functional status in aging populations and could inform the development of targeted intervention strategies aimed at preventing functional decline in older adults.

## Methods

### Participants and design

This study used data from the NHANES, a nationally representative cross-sectional survey conducted by the National Center for Health Statistics (NCHS) in the United States. NHANES is designed to assess the health and nutritional status of the noninstitutionalized civilian population through annual surveys of ∼5000 individuals. It collects comprehensive data, including demographic information, dietary intake, examination findings, laboratory results, and questionnaire responses. All survey design, methodology, and data are publicly available on the NHANES website (https://www.cdc.gov/nchs/nhanes/). Written informed consent was obtained from all participants, and the study protocol was approved by the Research Ethics Review Board of the NCHS [27].

A total of 101,316 participants from 10 consecutive NHANES cycles (1999–2018) were initially enrolled in this study. We included individuals aged ≥60 y with complete data on BRI, FI, and inflammatory markers.

### Functional impairment

FI was assessed based on self-reported difficulty in performing daily activities, using a 4-point Likert scale with responses ranging from 1 to 4 (1 = no difficulty, 2 = some difficulty, 3 = much difficulty, and 4 = unable to do). Physical limitations (PL) were evaluated through questions regarding the ability to: walk a quarter mile; climb 10 stairs; bend, squat, or kneel; lift or carry a 10-pound object; walk between rooms on the same floor; and stand up from an armless chair. Participants who responded with any difficulty other than “no difficulty” to at least one of these items were classified as having PL. BADL were assessed using questions on difficulty with getting in and out of bed; using a knife, fork, or drinking from a cup; standing for long periods; and dressing oneself. As NHANES does not include items on bathing or toileting, these domains were not captured. Difficulty with any of the above activities was considered indicative of BADL limitation. Instrumental activities of daily living (IADL) were assessed based on self-reported difficulty with managing finances, performing housework, and preparing meals. A positive response to any of these items was classified as an IADL limitation [28]. Consistent with previous research [29], FI was defined as the presence of any difficulty in at least one domain (PL, BADL, or IADL). This operational definition is widely used in survey-based epidemiological studies, including prior NHANES analyses, to identify disability-related functional limitations. It should be distinguished from the frailty index or frailty phenotypes (e.g., Fried criteria), which typically incorporate additional dimensions such as exhaustion, unintentional weight loss, low physical activity, and objective measures of strength and mobility. Although FI and frailty are related constructs commonly associated with aging, FI, as defined in this study focuses specifically on self-reported task-based difficulty rather than multisystem physiological decline.

### Body roundness index

The BRI was calculated using WC (cm) and height (cm), both of which were measured at mobile examination centers (MEC) following standardized protocols. To examine the association between BRI and FI, BRI values were categorized into 4 groups based on the 25th, 50th, and 75th percentiles. The BRI was derived using the following formula [12]:

$$\text{BRI}=364.2-365.5\times \sqrt{1-{\left(\frac{\text{WC}÷2\pi }{0.5\times H}\right)}^{2}}.$$

### Systemic inflammation response index

The SIRI was calculated using CBC data, specifically the counts of monocytes, neutrophils, and lymphocytes. The index was derived as follows: $\text{SIRI}=\frac{N\times M}{L}$, where N, M, and L represent neutrophil, monocyte, and lymphocyte counts, respectively [18]. In NHANES, CBC parameters were obtained using the Beckman Coulter method, which employs an automated dilution and mixing system for sample processing and a single-beam photometer for hemoglobin measurement. Leukocyte classification was performed using volume, conductivity, and scatter technology. All CBC measurements were conducted on blood samples at the MEC using the Beckman Coulter DxH 800 analyzer, providing comprehensive blood cell profiles for all participants.

### Covariates

Potential confounding factors for FI in older adults included demographic characteristics, smoking and alcohol consumption status, and history of chronic diseases. Demographic variables comprised age (<75, ≥75 y), sex (male, female), race/ethnicity (Mexican American, other Hispanic, non-Hispanic White, non-Hispanic Black, other race—including multiracial), educational level (less than high school, high school or above), marital status (married, cohabiting with partner, widowed, divorced, separated, or never married), and the poverty-to-income ratio (PIR). PIR was categorized into 3 levels (<1.3, 1.3–<3.5, and ≥3.5), with higher values indicating better family economic status [30]. In addition, smoking status (defined as having smoked ≥100 cigarettes in a lifetime), alcohol consumption (defined as having consumed ≥12 drinks of any alcoholic beverage in a given year), and health insurance coverage were also included. A self-reported questionnaire was administered to ascertain participants’ history of hypertension, diabetes, arthritis, congestive heart failure, coronary artery disease (CAD), stroke, COPD, kidney failure, and cancer.

### Statistical analysis

On the basis of the quartiles of the BRI, participants were categorized into 4 groups: Q1 (<4.51), Q2 (4.51–5.70), Q3 (5.70–7.07), and Q4 (>7.07). Categorical variables were presented as frequencies (weighted percentages) and compared across groups using the χ^2^test. Continuous variables with a normal distribution were expressed as means (SD) and compared using analysis of variance. Non-normally distributed continuous variables were reported as medians (IQR) and compared using the Kruskal–Wallis test. Weighted multivariate logistic regression models were used to examine the associations of BRI and the SIRI with the frailty index. Both continuous and quartile forms of BRI and SIRI were analyzed. To test for linear trends across quartiles, each quartile was assigned its median value, and this continuous variable was entered into the logistic regression model to derive the *P*-trend value. Two regression models were constructed: model A was unadjusted, and model B adjusted for age, sex, race or ethnicity, marital status, PIR, education level, smoking status, drinking status, hypertension, diabetes, CAD, stroke, COPD, kidney failure, and cancer. Before multivariable adjustment, multicollinearity among covariates was assessed using the variance inflation factor (VIF). Dose-response relationships between BRI, SIRI, and FI were evaluated using restricted cubic spline (RCS) regression with 4 knots. Subgroup analyses were conducted to explore these associations across strata defined by age (<75 y compared with ≥75 y), sex (male compared with female), drinking status (≥12 drinks per year compared with <12 drinks per year), smoking status (≥100 cigarettes per lifetime compared with <100 cigarettes per lifetime), and history of hypertension, CAD, and diabetes (yes compared with no). Interaction terms were introduced into the fully adjusted models to test for effect modification. Mediation analysis was performed to investigate the potential mediating role of SIRI in the relationship between BRI and FI. The bootstrap method with 5000 resamples was used to estimate 95% confidence intervals (CIs). In this analysis, BRI served as the independent variable, SIRI as the mediator, and FI as the dependent variable. Several sensitivity analyses were conducted. First, after excluding participants with missing covariate data, we re-examined the associations of BRI and SIRI with FI, as well as the mediating effect of SIRI. Second, logistic regression was used to assess the associations of BRI and SIRI with 3 distinct FI (PL, BADL, and IADL), and the mediating role of SIRI in these relationships was evaluated. Third, WC was used in place of BRI to compare the associations of different abdominal obesity indicators with FI and to reassess the mediating effect of SIRI. To ensure national representativeness, all analyses accounted for the complex survey design of the NHANES by incorporating the recommended sampling weights. Statistical analyses were performed using R software (version 4.4.1). RCS curves were generated using the “rcs” package, and mediation analyses were conducted using the “mediation” package. All tests were 2-sided, and statistical significance was defined as *P* < 0.05.

## Results

### Demographic characteristics

**FIGURE 1:**
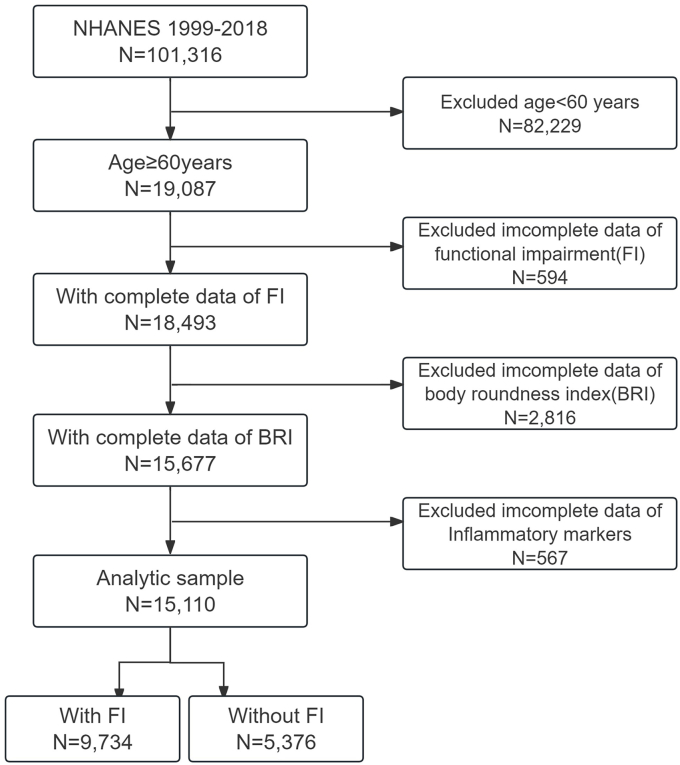
Selection of participants for our study from NHANES 1999–2018.

According to the inclusion criteria, a total of 82,229 participants were excluded due to age <60 y. Additional exclusions were made for individuals with missing data on FI (*n* = 594), BRI (*n* = 2816), or inflammatory markers (*n* = 567). The final analytic sample comprised 15,110 participants (Figure 1). As presented in Table 1, the median age (IQR) of the study population was 69 (64, 77) y. Among these participants, 9734 (64.4%) exhibited FI. Specifically, impairments in PL, BADL, and IADL were reported by 8871 (59.1%), 6962 (46.5%), and 4097 (28.3%) participants, respectively. The prevalence of FI, as well as impairments in PL, BADL, and IADL, varied significantly across BRI quartile groups (all *P* < 0.001). Furthermore, statistically significant differences were observed among BRI groups for all inflammatory markers, including lymphocyte, neutrophil, and monocyte counts, as well as the derived SIRI (all *P* < 0.001). In addition, participant characteristics such as age, sex, race/ethnicity, education level, marital status, PIR, BMI, alcohol consumption, and the prevalence of several chronic conditions (including arthritis, congestive heart failure, CAD, stroke, COPD, hypertension, diabetes, and kidney failure) also demonstrated significant differences across the BRI groups (all *P* < 0.001).

**TABLE 1:** Characteristics of study participants stratified by BRI levels, NHANES 1999–2018 (*n* = 15110).

Characteristic	Total participants	Q1	Q2	Q3	Q4	*P* value
	(*n* = 15110)	(*n* = 3776)	(*n* = 3780)	(*n* = 3776)	(*n* = 3778)	
Age (y), median (IQR)	69 (64, 77)	69 (63, 78)	70 (64, 77)	70 (64, 77)	69 (64, 75)	<0.001
Age group [*n* (%)]						<0.001
<75	10,375 (68.7)	2522 (66.8)	2567 (67.9)	2519 (66.7)	2767 (73.2)	
≥75	4735 (31.3)	1254 (33.2)	1213 (32.1)	1257 (33.3)	1011 (26.8)	
Sex [*n* (%)]						<0.001
Male	7443 (49.3)	1967 (52.1)	2139 (56.6)	1892 (50.1)	1445 (38.2)	
Female	7667 (50.7)	1809 (47.9)	1641 (43.4)	1884 (49.9)	2333 (61.8)	
Race/ethnicity [*n* (%)]						<0.001
Mexican American	2269 (15.0)	335 (8.9)	562 (14.9)	670 (17.7)	702 (18.6)	
Other Hispanic	1168 (7.7)	200 (5.3)	306 (8.1)	323 (8.6)	339 (9.0)	
Non-Hispanic White	7831 (51.8)	2047 (54.2)	1973 (52.2)	1939 (51.4)	1872 (49.6)	
Non-Hispanic Black	2868 (19.0)	806 (21.3)	658 (17.4)	657 (17.4)	747 (19.8)	
Other race-including multiracial	974 (6.4)	388 (10.3)	281 (7.4)	187 (5.0)	118 (3.1)	
Educational level [*n* (%)]						<0.001
<High school graduate	5037 (33.4)	1044 (27.8)	1225 (32.4)	1325 (35.2)	1443 (38.2)	
≥High school graduate	10,042 (66.6)	2718 (72.2)	2551 (67.6)	2443 (64.8)	2330 (61.8)	
Marital status [*n* (%)]						<0.001
Married/living with partner	8867 (59.2)	2234 (59.9)	2364 (63.0)	2267 (60.4)	2002 (53.5)	
Widowed/divorced/separated/never married	6106 (40.8)	1495 (40.1)	1387 (37.0)	1485 (39.6)	1739 (46.5)	
Family poverty income ratio [*n* (%)]						<0.001
<1.3	3917 (28.8)	868 (25.6)	899 (26.3)	1011 (29.5)	1139 (33.7)	
1.3–3.5	5789 (42.5)	1393 (41.1)	1443 (42.3)	1475 (43.0)	1478 (43.8)	
≥3.5	3900 (28.7)	1126 (33.2)	1070 (31.4)	943 (27.5)	761 (22.5)	
Covered by health insurance [*n* (%)]	13,916 (92.4)	3513 (93.4)	3473 (92.3)	3472 (92.3)	3458 (91.9)	0.086
Alcohol user [*n* (%)]	9710 (66.9)	2503 (69.3)	2535 (69.7)	2406 (66.2)	2266 (62.3)	<0.001
Smoker [*n* (%)]	7785 (51.6)	1970 (52.3)	1950 (51.6)	1954 (51.8)	1911 (50.6)	0.527
Arthritis [*n* (%)]	7494 (49.7)	1556 (41.3)	1684 (44.7)	1932 (51.4)	2322 (61.6)	<0.001
Congestive heart failure, [*n* (%)]	1048 (7.0)	184 (4.9)	206 (5.5)	259 (6.9)	399 (10.6)	<0.001
CAD [*n* (%)]	1494 (10.0)	289 (7.7)	402 (10.7)	379 (10.1)	424 (11.4)	<0.001
Stroke [*n* (%)]	1126 (7.5)	255 (6.8)	242 (6.4)	295 (7.8)	334 (8.9)	<0.001
COPD [*n* (%)]	1157 (7.7)	244 (6.5)	229 (6.1)	270 (7.2)	414 (11.0)	<0.001
Cancer diagnosis [*n* (%)]	2899 (19.2)	767 (20.3)	720 (19.1)	739 (19.6)	673 (17.8)	0.045
Hypertension [*n* (%)]	8881 (58.9)	1697 (45.1)	2091 (55.4)	2389 (63.4)	2704 (71.7)	<0.001
Diabetes mellitus [*n* (%)]	3379 (23.1)	435 (11.8)	674 (18.4)	935 (25.7)	1335 (37.0)	<0.001
Failing kidney [*n* (%)]	768 (5.1)	138 (3.7)	158 (4.2)	207 (5.5)	265 (7.0)	<0.001
PL [*n* (%)]	8871 (59.1)	1742 (46.5)	1943 (51.6)	2307 (61.5)	2879 (76.8)	<0.001
BADL [*n* (%)]	6962 (46.5)	1361 (36.4)	1501 (40.1)	1773 (47.4)	2327 (62.3)	<0.001
IADL [*n* (%)]	4097 (28.3)	820 (22.5)	821 (22.6)	1027 (28.6)	1429 (39.8)	<0.001
FI [*n* (%)]	9734 (64.4)	2015 (53.4)	2187 (57.9)	2511 (66.5)	3021 (80.0)	<0.001
Lymphocyte, median (IQR)	1.9 (1.5, 2.4)	1.8 (1.4, 2.2)	1.9 (1.5, 2.4)	1.9 (1.5, 2.4)	2 (1.6, 2.5)	<0.001
Neutrophil, median (IQR)	4 (3.1, 5.0)	3.7 (2.8, 4.7)	3.9 (3.1, 4.8)	4 (3.2, 5.0)	4.3 (3.4, 5.3)	<0.001
Monocyte, median (IQR)	0.6 (0.4, 0.7)	0.5 (0.4, 0.7)	0.6 (0.4, 0.7)	0.6 (0.5, 0.7)	0.6 (0.5, 0.7)	<0.001
SIRI, median (IQR)	1.1 (0.8, 1.7)	1.1 (0.7, 1.6)	1.1 (0.8, 1.6)	1.2 (0.8, 1.7)	1.2 (0.8, 1.8)	<0.001

Abbreviations: BADL, basic activities of daily living; BRI, body roundness index; CAD, coronary artery disease; COPD, chronic obstructive pulmonary disease; FI, functional impairment; IADL, instrumental activities of daily living; PL, physical limitation; SIRI, systemic inflammation response index.

### Relationship between BRI and FI

As shown in Table 2, in the unadjusted model (model A), higher BRI was consistently associated with increased odds of FI. Multicollinearity diagnostics revealed that all covariates had VIF values <2.5 (Supplementary Table 1), indicating no significant multicollinearity and supporting their inclusion in the adjusted model. In the fully adjusted model, each SD increase in BRI was associated with a 1.70-fold increase in the odds of FI. Similarly, relative to participants in the lowest BRI quartile (Q1), those in the highest quartile (Q4) exhibited significantly elevated odds of FI (*P*-trend < 0.001).

**TABLE 2:** Associations between BRI and function impairment among United States older adults^1^.

	Model A			Model B		
	OR	95% CI	*P* value	OR	95% CI	*P* value
Continuous (per SD)	1.79	1.69, 1.89	<0.001	1.70	1.60, 1.80	<0.001
Quartiles						
Q1	Reference	—	—	Reference	—	—
Q2	1.33	1.16, 1.53	<0.001	1.37	1.19, 1.59	<0.001
Q3	1.79	1.56, 2.06	<0.001	1.68	1.45, 1.95	<0.001
Q4	4.23	3.66, 4.88	<0.001	3.65	3.14, 4.24	<0.001
*P-*trend	—	—	<0.001	—	—	<0.001

Abbreviations: BRI: body roundness index; CI, confidence interval; OR, odds ratio.

1 The associations between BRI and function impairment among United States older adults are presented as ORs (95% CI). Model A did not adjust for any covariates. Model B adjusted for age, sex, race or ethnicity, education level, marital status, alcohol use, smoking—cigarette use, and hypertension, diabetes, coronary artery disease.

**FIGURE 2:**
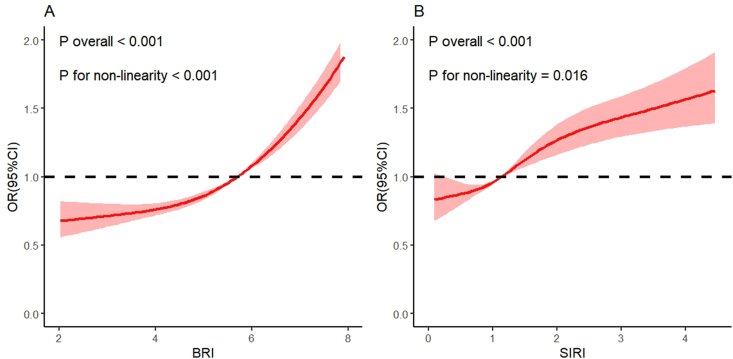
Dose-response relationship between BRI, SIRI, and FI. (A) BRI and FI. (B) SIRI and FI. BRI, body roundness index; CI, confidence interval; FI, functional impairment; OR, odds ratio; SIRI, Systemic Inflammation Response Index.

**FIGURE 3:**
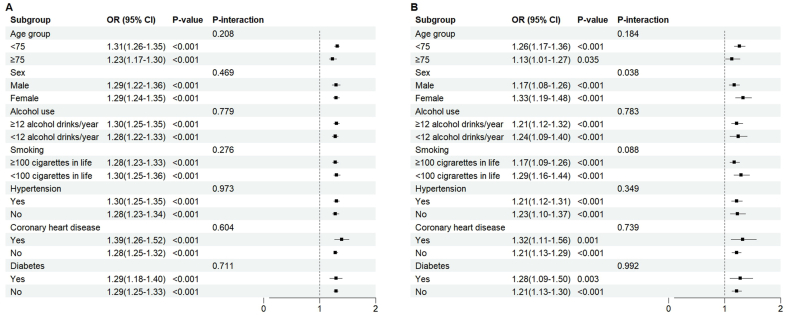
Subgroup analysis of the association between BRI, SIRI, and FI. (A) BRI and FI. (B) SIRI and FI. BRI, body roundness index; CI, confidence interval; FI, functional impairment; OR, odds ratio; SIRI, Systemic Inflammation Response Index.

RCS analysis revealed a positive, nonlinear dose–response relationship between BRI and the odds of FI (*P*-overall < 0.001; *P*-nonlinearity < 0.001) (Figure 2A). This nonlinear association remained consistent in subgroup analyses stratified by sex (Supplementary Figure 1). In the subgroup analyses, higher BRI was significantly associated with increased odds of FI across all examined strata, and no significant interactions were observed between BRI and any of the covariates (Figure 3A).

### Relationship between SIRI and FI

As presented in Table 3, in the unadjusted model (model A), when SIRI was modeled as a continuous variable, each SD increase was associated with a 1.30-fold increase in the odds of FI. When SIRI was examined categorically by quartiles, participants in the highest quartile (Q4) exhibited significantly elevated odds of FI compared with those in the lowest quartile (Q1) (*P*-trend < 0.001). In the fully adjusted model, each SD increment in SIRI remained significantly associated with increased odds of FI (odds ratio: 1.23). Consistently, participants in the highest SIRI quartile continued to demonstrate significantly higher odds of FI relative to the lowest quartile (*P*-trend < 0.001).

**TABLE 3:** Associations between SIRI and function impairment among United States older adults^1^.

	Model A			Model B		
	OR	95% CI	*P* value	OR	95% CI	*P* value
Continuous (per SD)	1.30	1.23, 1.37	<0.001	1.23	1.16, 1.32	<0.001
Quartiles						
Q1	Reference	—	—	Reference	—	—
Q2	1.09	0.97, 1.23	0.143	1.10	0.95, 1.27	0.194
Q3	1.41	1.21, 1.64	<0.001	1.36	1.14, 1.63	<0.001
Q4	1.78	1.53 2.08	<0.001	1.63	1.36, 1.96	<0.001
*P*-trend	—	—	<0.001	—	—	<0.001

Abbreviations: CI, confidence interval; OR, odds ratio; SIRI: Systemic Inflammation Response Index.

1 The associations between BRI and function impairment among United States older adults are presented as ORs (95% CI). Model A did not adjust for any covariates. Model B adjusted for age, sex, race/ethnicity, education level, marital status, alcohol use, smoking—cigarette use, and hypertension, diabetes, coronary artery disease.

RCS analysis revealed a positive, nonlinear dose–response relationship between SIRI and the odds of FI (*P*-overall < 0.001; *P*-nonlinearity = 0.016) (Figure 2B). This nonlinear association remained consistent in subgroup analyses stratified by sex (Supplementary Figure 1).

In subgroup analyses, higher SIRI was significantly associated with increased odds of FI across all examined strata. A significant interaction was observed between SIRI and sex (*P*-interaction = 0.038) (Figure 3B).

### Mediation analysis

**FIGURE 4:**
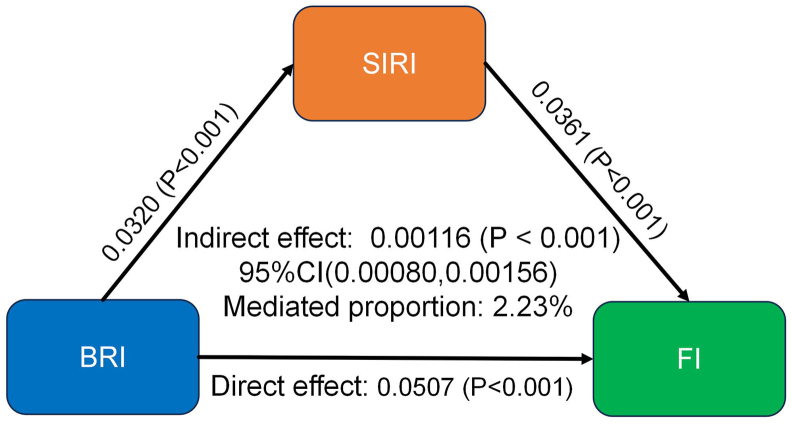
Mediating role of SIRI in the relationship between BRI and FI. BRI, body roundness index; FI, functional impairment; SIRI, Systemic Inflammation Response Index.

After adjusting for all covariates, mediation analysis revealed that SIRI significantly mediated the relationship between BRI and FI. The indirect effect of BRI on FI through SIRI was 0.00116 (95% CI: 0.00080, 0.00156; *P* < 0.001), accounting for 2.23% of the total effect (Figure 4).

### Sensitivity analysis

In the fully adjusted model restricted to participants with complete covariate data, each SD increase in BRI remained significantly associated with a 1.24-fold increase in the odds of FI. Consistently, participants in the highest BRI quartile (Q4) exhibited significantly elevated odds of FI compared with those in the lowest quartile (Q1) (*P*-trend < 0.001) (Supplementary Table 2). Similarly, each SD increment in SIRI was associated with a 1.17-fold increase in the odds of FI, and participants in the highest SIRI quartile demonstrated significantly higher odds of FI (*P*-trend < 0.001) (Supplementary Table 3). Mediation analysis further confirmed that SIRI significantly mediated the relationship between BRI and FI, with an indirect effect of 0.00088, accounting for 1.66% of the total effect (Supplementary Figure 2).

In the fully adjusted models, BRI was significantly and positively associated with impairments in PL, BADL, and IADL. Similarly, SIRI demonstrated significant positive associations with all 3 FI outcomes. Mediation analyses revealed that SIRI significantly mediated the relationships between BRI and each FI measure. The indirect effect of BRI on PL through SIRI was 0.00127, accounting for 2.15% of the total effect (Supplementary Figure 3). For BADL, the indirect effect was 0.00146, representing 2.72% of the total effect (Supplementary Figure 4). For IADL, the indirect effect was 0.00120, comprising 3.37% of the total effect (Supplementary Figure 5).

In the fully adjusted model using WC as an alternative abdominal obesity indicator, each SD increase in WC was associated with a 1.67-fold increase in the odds of FI. Consistently, participants in the highest WC quartile (Q4) exhibited significantly elevated odds of FI compared with those in the lowest quartile (Q1) (*P*-trend < 0.001) (Supplementary Table 4). Mediation analysis further demonstrated that SIRI significantly mediated the relationship between WC and FI, with an indirect effect of 0.00013, accounting for 1.78% of the total effect (Supplementary Figure 6).

## Discussion

To the best of our knowledge, this study is the first to investigate the associations of BRI and SIRI with FI in a large, representative cohort of older adults. Our findings demonstrate that higher BRI is significantly associated with increased odds of FI, and that elevated SIRI levels are similarly linked to higher FI odds. Notably, SIRI was found to partially mediate the relationship between BRI and FI, suggesting a potential inflammatory pathway linking abdominal obesity to frailty. The robustness of these findings was further supported by comprehensive subgroup and sensitivity analyses.

This study is the first to establish a significant positive association between BRI and FI in a large, representative sample of older adults. Notably, our operational definition of FI, based on self-reported FIs across multiple domains, differs from validated frailty indices that typically integrate clinical signs, symptoms, and objective performance measures. Although FI and frailty are conceptually overlapping constructs—both predictive of adverse health outcomes—they are not interchangeable [29]. Frailty indices assess physiological reserve depletion and systemic vulnerability through the accumulation of multidimensional health deficits [31], whereas our FI measures more directly capture individuals’ perceived difficulties in performing daily activities. Importantly, both constructs serve as core predictors of adverse outcomes in older populations. Theou et al. [32] demonstrated that despite methodological heterogeneity, various frailty concepts consistently predict adverse health outcomes, supporting the validity of diverse operational approaches. Furthermore, both FI and established frailty indices are influenced by shared pathophysiological pathways, particularly chronic inflammation [24,33]. Building upon this operational definition, our findings demonstrate consistency with the broader literature on obesity-related functional decline. Future studies are warranted to examine whether the associations of BRI and SIRI with FI extend to frailty measured by established indices, such as the cumulative deficit model or the Fried phenotype. Such comparative investigations may yield additional insights into the biological pathways underlying functional decline and help contextualize our findings within the wider frailty research framework. Our findings are consistent with prior studies examining obesity-related functional decline. Sternfeld et al. [34] in a cross-sectional study of 1655 older adults in Sonoma, California, reported that higher body fat levels were associated with increased risk of functional limitations. Himes et al. [35] found that obesity prevalence increased with age among adults aged ≥70 y, with obesity most strongly associated with activities of daily living (ADL) limitations and activity-related difficulties in females. Similarly, Tareque et al. [36] demonstrated that higher BMI correlated with greater ADL and IADL impairment. Using NHANES data from 2005 to 2010, Batsis et al. [37] examined 4976 adults aged ≥60 y and found that elevated WC was associated with increased risk of PL and ADL disability. Notably, most previous investigations used traditional anthropometric measures such as overall adiposity, BMI, or WC as independent variables. The present study extends this literature by introducing BRI—a novel anthropometric index specifically designed to assess central obesity [12]—as a predictor of FI. Higher BRI reflects greater visceral fat accumulation, which may predispose individuals to cardiovascular disease [38], diabetes [39], and other chronic conditions, collectively contributing to functional decline. Thus, our findings provide important complementary evidence to the existing literature. Recent evidence directly supports the observed BRI–FI association. A study using NHANES data [40] demonstrated that higher BRI was significantly and positively associated with increased frailty prevalence in older adults, providing direct external validation for our results. The underlying mechanism may involve chronic inflammation. Cervellati et al. [41] reported that systemic low-grade inflammation, evidenced by elevated high-sensitivity-CRP (hs-CRP), was independently associated with functional disability in older patients with dementia, suggesting inflammation as a key pathophysiological pathway linking adiposity to FI. Pan et al. [42] systematically reviewed the association between inflammatory markers and physical decline, identifying chronic low-grade inflammation as a core biological mechanism in aging, with IL-6, CRP, and TNF-α significantly associated with deterioration. Their work provides a theoretical framework for the present study. The current investigation advances this field by jointly modeling BRI and SIRI, a composite inflammatory index, and formally testing SIRI as a mediator in the central obesity-FI relationship—a hypothesized pathway that has received limited empirical examination using composite inflammatory biomarkers.

Our results further demonstrate a positive association between SIRI, a novel composite inflammatory marker, and FI in older adults. These findings are consistent with and extend previous research on inflammation-related functional decline. Klausen et al. [43] studied older emergency department patients and found that elevated inflammatory biomarkers, including soluble urokinase plasminogen activator receptor (suPAR) and TNF-α, were associated with increased risk of physical and organ dysfunction, with suPAR potentially serving as a key mediator linking organ dysfunction to physical impairment. Penninx et al. [44] in a cross-sectional study of 2979 adults aged 70–79 y from Pittsburgh and Memphis, reported that higher concentrations of IL-6, TNF-α, and CRP were associated with increased incidence of activity limitations. Similarly, Collerton et al. [45] examined 845 adults aged ≥85 y in Newcastle and confirmed that elevated inflammatory markers, including IL-6, TNF-α, CRP, and neutrophils, were associated with increased frailty risk. The biological plausibility of these associations is supported by mechanistic evidence. Older adults with elevated inflammatory markers may exhibit persistent immune system activation, contributing to chronic inflammation in joints and other tissues. This process can lead to cartilage and bone damage, induce pain, precipitate mobility problems, and ultimately contribute to FI [46]. Elevated SIRI levels reflect systemic inflammation and immune dysregulation, which are implicated in the pathogenesis, progression, and poor prognosis of multiple age-related conditions, including cardiovascular disease [20], COPD [21], malignancies [22], and cognitive impairment [47]. To our knowledge, this study is the first to identify a significant association between SIRI and FI in a large, representative sample of older adults. These findings suggest that SIRI may serve as a valuable biomarker for identifying older adults at risk of functional decline, with potential applications in clinical screening and risk stratification. Future research should prospectively validate these findings and explore the utility of SIRI in guiding targeted interventions to prevent or delay FI in aging populations.

A notable finding of this study is that SIRI partially mediated the relationship between BRI and FI in older adults. This mediating role is supported by prior evidence linking obesity to systemic inflammation. Zhou et al. [48] demonstrated that higher obesity levels predicted elevated SIRI in US adults. Similarly, a cross-sectional study [49] reported that increased BMI was associated with higher white blood cell, neutrophil, lymphocyte, and platelet counts, as well as elevated Systemic Immune-Inflammation Index levels. The biological underpinnings may involve hypoxia in hypertrophic adipose tissue, which stimulates inflammatory gene expression and activates immune cells. Notably, visceral adipose tissue and central obesity exert a greater influence on inflammatory processes than total body fat [50], providing a mechanistic basis for the BRI–SIRI relationship observed in our study.

Regarding the SIRI–FI association, accumulating evidence supports the role of inflammation in functional decline. A multicenter retrospective study demonstrated that SIRI serves as a prognostic biomarker in rheumatoid arthritis, with higher levels indicating more severe joint structural damage, poorer prognosis, and increased muscle loss, collectively contributing to mobility limitations [51]. Among elderly hypertensive patients, elevated SIRI levels are associated with increased risk of osteoporosis and fractures, leading to behavioral restrictions and reduced physical activity [52]. Aiello et al. [24], in a cross-sectional study of community-dwelling older adults in Latin America, found that higher CRP concentrations correlated with greater ADL and IADL impairment. Another study of 739 community-dwelling older adults confirmed that elevated CRP was independently associated with increased frailty risk [25]. The pathophysiological pathways linking inflammation to FI are multifaceted. Systemic inflammation may affect physical function through multiple physiological systems, including musculoskeletal [53], nervous [54] and cardiovascular [55], mechanisms, collectively contributing to functional decline. These studies provide robust theoretical support for our findings. It is noteworthy that although statistically significant, the mediating effect of SIRI was relatively modest, accounting for 2.23% of the total effect. This magnitude is consistent with previous investigations examining SIRI as a mediator in other health outcomes. Tang et al. [56] reported that SIRI mediated 1.65% of the association between hypertension and stroke. Similarly, Tang et al. [57] found that SIRI mediated 5.8% of the relationship between cardiovascular health and severe abdominal aortic calcification. The modest mediation proportion likely reflects the multifaceted nature of obesity-related functional decline. The pathophysiological pathways linking central obesity, as measured by BRI, to FI are numerous and complex, with chronic low-grade inflammation representing one of several parallel mechanisms [42]. Beyond inflammation, insulin resistance, hormonal alterations, and psychosocial factors may independently or interactively contribute to functional decline [58]. Therefore, future investigations should adopt a multi-target approach to elucidate the relative contributions of each pathway, utilizing more specific biomarkers and longitudinal designs to advance our understanding of obesity-related FI.

From a clinical perspective, both BRI and SIRI offer practical advantages in geriatric care. Derived from routine physical examination data, these indicators are objective, cost-effective, and readily accessible, rendering them suitable for broad application in community and clinical settings. Although they are not intended to replace comprehensive geriatric assessment, their combined use may serve as a powerful initial screening and risk stratification tool for FI in older adults [59]. This approach facilitates early identification of at-risk individuals and provides objective evidence to inform the development of multidimensional, personalized intervention strategies targeting nutrition, physical activity, and inflammation management. Ultimately, such evidence-based preventive strategies support a paradigm shift from reactive to proactive management in aging populations.

This study offers several novel insights into the association between central obesity and functional decline in older adults, with notable strengths. First, the analysis was conducted in a relatively large, nationally representative sample, enhancing the generalizability of the findings. Second, comprehensive adjustment for potential confounders strengthened the validity of the observed associations. Third, the use of BRI, an anthropometric index derived from objective measurements, provided a standardized assessment of central adiposity. However, several limitations warrant consideration. First, the cross-sectional design precludes causal inference regarding the relationships among BRI, SIRI, and FI. Longitudinal studies are needed to establish temporal sequences and elucidate causal pathways. Second, FI was assessed primarily through self-reported data, which may be subject to reporting bias. Future studies incorporating objective functional assessments would help validate and extend our findings. Third, SIRI, although derived from routine CBC data, is not a clinically validated diagnostic tool. Its value may be influenced by age, sex [60], and underlying conditions unrelated to chronic inflammation, such as acute infections or noninflammatory chronic diseases [61]. Compared with well-validated inflammatory biomarkers like hs-CRP or IL-6, SIRI lacks established population-based reference ranges and longitudinal validation in aging cohorts. Nevertheless, its derivation from widely available and low-cost laboratory data offers practical advantages for epidemiological research and potential clinical screening applications. Fourth, the clinical predictive utility of BRI relative to traditional anthropometric indicators warrants careful consideration. Pezeshki et al. [62] reported that although novel indices such as BRI demonstrate predictive value in certain populations, they do not significantly outperform traditional measures, including BMI, WC, or waist-to-height ratio (WHtR) in discriminating hypertension risk. A systematic review on hypertension prediction [63] further confirmed that although BRI exhibits discriminative ability comparable to BMI, it does not substantially surpass WC or WHtR in predicting cardiometabolic diseases. These observations suggest that the clinical advantage of BRI as an independent predictive tool may be limited. In summary, while our findings contribute to understanding the obesity-inflammation-disability pathway using emerging indices, they should be interpreted with awareness of the comparative limitations of these measures. Further validation against reference-standard clinical assessments and established biomarkers is warranted to substantiate and extend our observations.

Finally, the study population was limited to older adults in the United States, which may restrict the generalizability of our findings to other racial/ethnic groups or geographic regions with different sociocultural and environmental contexts. Therefore, replication of these findings in diverse international cohorts is warranted to establish the cross-population validity of the observed associations.

In conclusion, higher BRI levels are significantly associated with elevated odds of FI in older adults, with the systemic inflammatory marker SIRI partially mediating this relationship. These findings offer a novel perspective on the link between visceral obesity and functional decline in aging populations and may inform early, targeted prevention strategies for FI in older adults.

## Author contributions

The authors’ responsibilities were as follows – MZ: contributed to conceptualization, formal analysis, writing—original draft; MZ, CM, HQ, YY: contributed to writing—review and editing; MZ, CM: contributed to methodology; CM, HQ: contributed to supervision; and all authors: read and approved the final manuscript.

## Data availability

The data in this study were downloaded from the official website of NHANES (https://www.cdc.gov/nchs/nhanes/), which is a publicly available dataset that is free of charge.

## Funding

This work was sponsored by the Quzhou City Science and Technology Research Guiding Project (2022038, 2024ZD095).

## Declaration of Generative AI and AI-assisted technologies in the writing process

We reported no use of any generative AI and AI-assisted technologies in the writing process.

## Conflict of interest

The authors report no conflicts of interest.
