# Supplementary material for: Association between Body Roundness Index and Functional Impairment in Older Adults: Exploring the Potential Mediating Role of Systemic Inflammation Response Index
Source: Curr Dev Nutr. 2026 Mar 12;10(4):107671. doi: 10.1016/j.cdnut.2026.107671 (PMC13068833; doi:10.1016/j.cdnut.2026.107671)
Supplement: multimedia component 1 [file mmc1.doc]

| **Supplementary Material**  **Association between Body Roundness Index and Functional Impairment in Older Adults: Exploring the Potential Mediating Role of Systemic Inflammation Response Index**  Min Zhou et al  Supplementary Table 1. Multicollinearity test results  Supplementary Table 2. Sensitivity Analysis of Associations between BRI and Function Impairment after excluding participants with missing covariates  Supplementary Table 3. Sensitivity Analysis of Associations between SIRI and Function Impairment after excluding participants with missing covariates  Supplementary Figure 1. Dose-response relationship in male and female subgroup  Supplementary Figure 2. Sensitivity Analysis of mediating role of SIRI in the relationship between BRI and FI after excluding participants with missing covariates  Supplementary Figure 3. Analysis of mediating role of SIRI in the relationship between BRI and PL  Supplementary Figure 4. Analysis of mediating role of SIRI in the relationship between BRI and BADL  Supplementary Figure 5. Analysis of mediating role of SIRI in the relationship between BRI and IADL  Supplementary Table 4. Associations between WC and FI among US older adults  Supplementary Figure 6. Analysis of mediating role of SIRI in the relationship between WC and FI   | Supplementary Table 1. Multicollinearity test results. | | | --- | --- | | Variable | VIF | | BRI | 1.137 | | SIRI | 1.120 | | Age group | 1.147 | | Sex | 1.322 | | Race/ethnicity | 1.435 | | Marital status | 1.141 | | Educational level | 1.185 | | Smoker | 1.175 | | Alcohol user | 1.210 | | Hypertension | 1.108 | | Diabetes mellitus | 1.115 | | CHD | 1.062 |   Abbreviations: BRI, body roundness index; CHD, Coronary Heart Disease; SIRI, Systemic Inflammation Response Index;VIF, Variance Inflation Factor.  Supplementary Table 2. Sensitivity Analysis of Associations between BRI and Function Impairment after excluding participants with missing covariates a.   |  |  | Model A |  |  | Model B |  | | --- | --- | --- | --- | --- | --- | --- | |  | OR | 95% CI | p-Value | OR | 95% CI | p-Value | | Continuous (per SD) | 1.27 | 1.23-1.31 | <0.001 | 1.24 | 1.20-1.28 | <0.001 | | Quartiles |  |  |  |  |  |  | | Q1 | Reference |  |  | Reference |  |  | | Q2 | 1.39 | 1.19-1.63 | <0.001 | 1.39 | 1.18-1.63 | <0.001 | | Q3 | 1.69 | 1.43-1.98 | <0.001 | 1.56 | 1.32-1.85 | <0.001 | | Q4 | 3.38 | 2.86-3.99 | <0.001 | 2.92 | 2.48-3.43 | <0.001 | | p-trend |  |  | <0.001 |  |  | <0.001 |   Abbreviations: BRI, body roundness index; CI, confidence interval; OR, odds ratio. ^a^ The associations between BRI and Function Impairment among US older adults are presented as ORs (95% CI). Model A did not adjust for any covariates. Model B adjusted for age, sex, race, education level, marital status, alcohol use, smoking—cigarette use, and hypertension, diabetes, coronary heart disease. |
| --- | --- | --- | --- | --- | --- | --- | --- | --- | --- | --- | --- | --- | --- | --- | --- | --- | --- | --- | --- | --- | --- | --- | --- | --- | --- | --- | --- | --- | --- | --- | --- | --- | --- | --- | --- | --- | --- | --- | --- | --- | --- | --- | --- | --- | --- | --- | --- | --- | --- | --- | --- | --- | --- | --- | --- | --- | --- | --- | --- | --- | --- | --- | --- | --- | --- | --- | --- | --- | --- | --- | --- | --- | --- | --- | --- | --- | --- | --- | --- | --- | --- | --- | --- | --- | --- | --- | --- | --- | --- | --- | --- |

| Supplementary Table 3. Sensitivity Analysis of Associations between SIRI and Function Impairment after excluding participants with missing covariates ^a^.   |  |  | Model A |  |  | Model B |  | | --- | --- | --- | --- | --- | --- | --- | |  | OR | 95% CI | p-Value | OR | 95% CI | p-Value | | Continuous (per SD) | 1.20 | 1.13-1.27 | <0.001 | 1.17 | 1.09-1.25 | <0.001 | | Quartiles |  |  |  |  |  |  | | Q1 | Reference |  |  | Reference |  |  | | Q2 | 1.07 | 0.93-1.23 | 0.376 | 1.06 | 0.91-1.24 | 0.455 | | Q3 | 1.25 | 1.05-1.50 | 0.014 | 1.25 | 1.03-1.51 | 0.021 | | Q4 | 1.52 | 1.28-1.80 | <0.001 | 1.44 | 1.18-1.74 | <0.001 | | p-trend |  |  | <0.001 |  |  | <0.001 |   Abbreviations: CI, confidence interval; OR, odds ratio; SIRI, Systemic Inflammation Response Index. ^a^ The associations between SIRI and Function Impairment among US older adults are presented as ORs (95% CI). Model A did not adjust for any covariates. Model B adjusted for age, sex, race, education level, marital status, alcohol use, smoking—cigarette use, and hypertension, diabetes, coronary heart disease. |
| --- | --- | --- | --- | --- | --- | --- | --- | --- | --- | --- | --- | --- | --- | --- | --- | --- | --- | --- | --- | --- | --- | --- | --- | --- | --- | --- | --- | --- | --- | --- | --- | --- | --- | --- | --- | --- | --- | --- | --- | --- | --- | --- | --- | --- | --- | --- | --- | --- | --- | --- | --- | --- | --- | --- | --- | --- | --- | --- | --- | --- | --- | --- | --- |


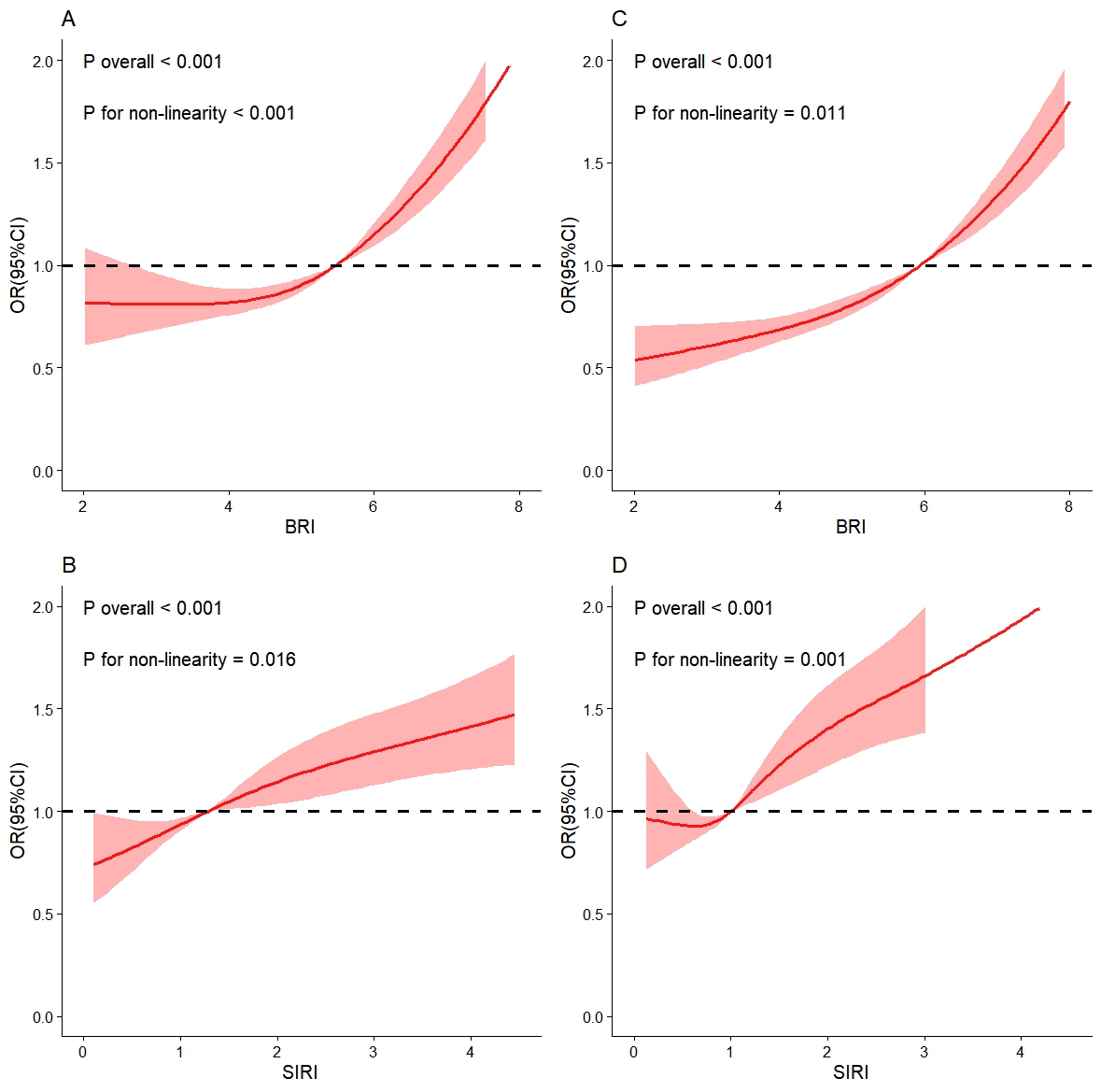


Supplementary Figure 1. Dose-response relationship in male and female subgroup. (A) BRI and FI in males. (B) SIRI and FI in males. (C) BRI and FI in females. (D) SIRI and FI in females. Abbreviations: BRI, body roundness index; FI, Functional Impairment; OR, odds ratio; SIRI, Systemic Inflammation Response Index.


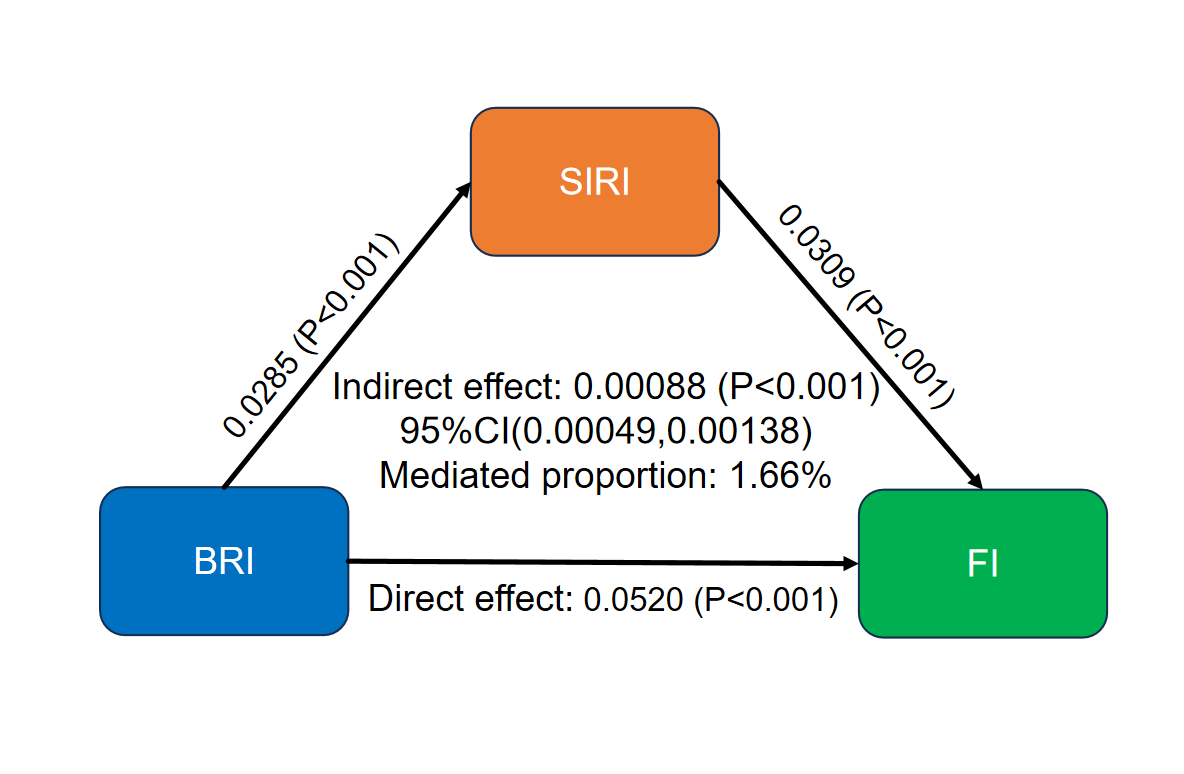


Supplementary Figure 2. Sensitivity Analysis of mediating role of SIRI in the relationship between BRI and FI after excluding participants with missing covariates.

Abbreviations: BRI, body roundness index; FI, Functional Impairment; SIRI, Systemic Inflammation Response Index.


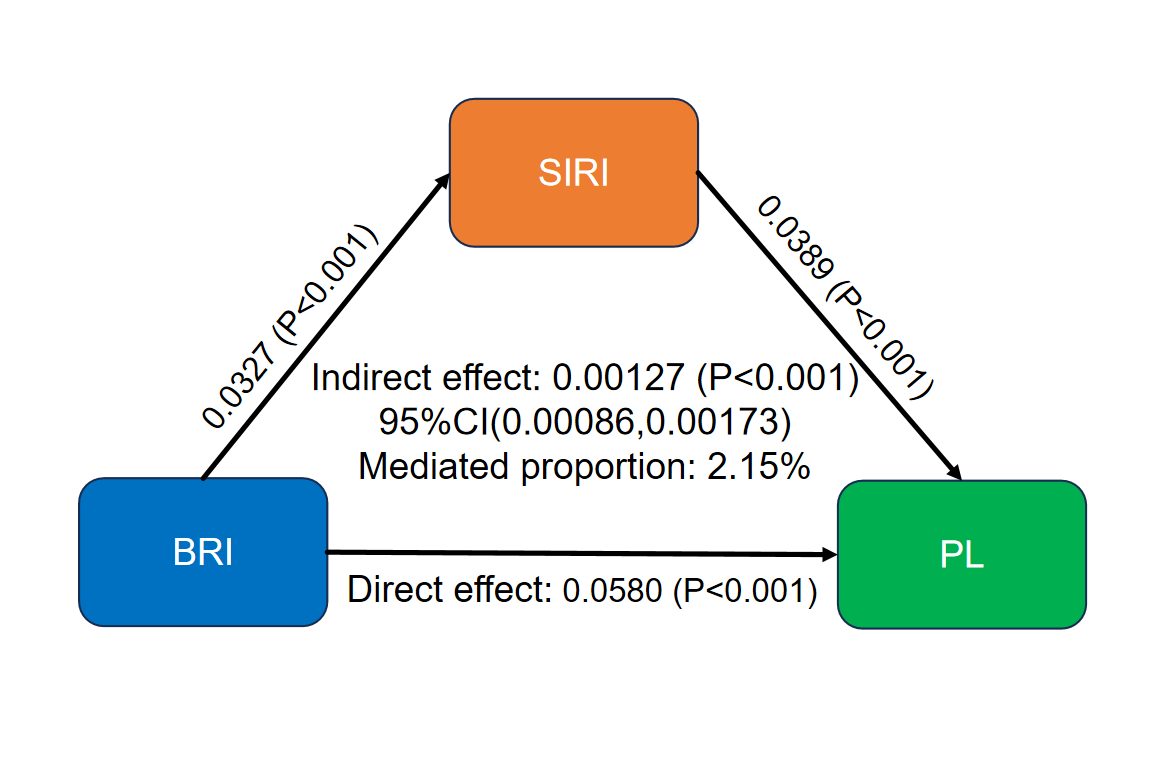


Supplementary Figure 3. Analysis of mediating role of SIRI in the relationship between BRI and PL.

Abbreviations: BRI, body roundness index; PL, Physical Limitation; SIRI, Systemic Inflammation Response Index.


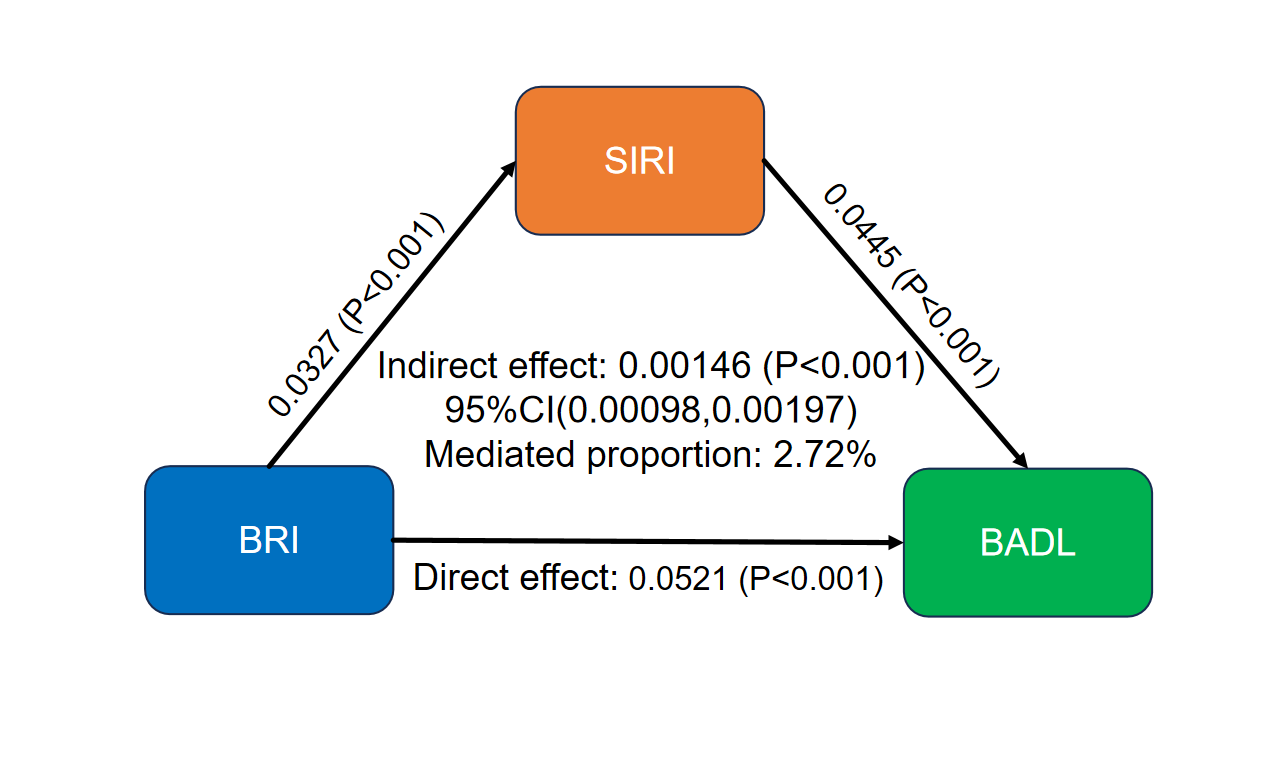


Supplementary Figure 4. Analysis of mediating role of SIRI in the relationship between BRI and BADL.

Abbreviations: BRI, body roundness index; BADL, Basic Activities of Daily Living; SIRI, Systemic Inflammation Response Index.


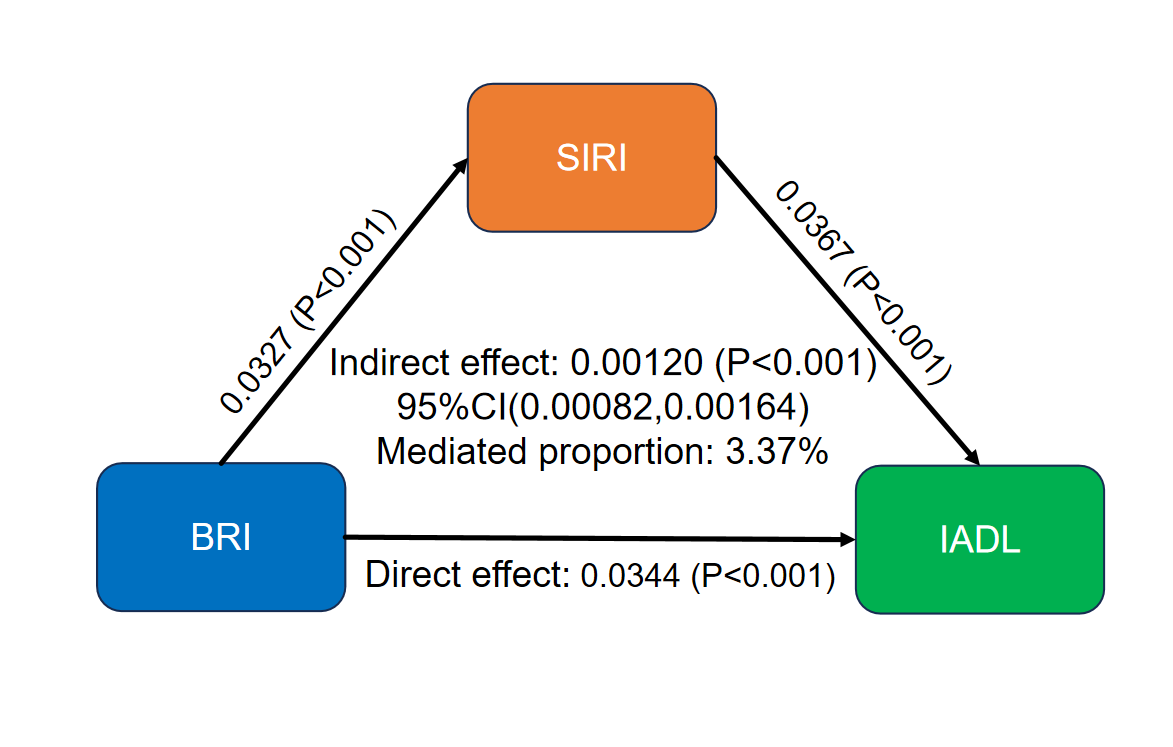


Supplementary Figure 5. Analysis of mediating role of SIRI in the relationship between BRI and IADL.

Abbreviations: BRI, body roundness index; IADL, Instrumental Activities of Daily Living; SIRI, Systemic Inflammation Response Index.

| Supplementary Table 4. Associations between WC and FI among US older adults ^a^. | | | | | | |
| --- | --- | --- | --- | --- | --- | --- |
|  |  | Model A |  |  | Model B |  |
|  | OR | 95% CI | p-Value | OR | 95% CI | p-Value |
| Continuous (per SD) | 1.50 | 1.42-1.57 | <0.001 | 1.67 | 1.58-1.76 | <0.001 |
| Quartiles |  |  |  |  |  |  |
| Q1 | Reference |  |  | Reference |  |  |
| Q2 | 1.18 | 1.03-1.35 | 0.015 | 1.36 | 1.18-1.57 | <0.001 |
| Q3 | 1.52 | 1.32-1.74 | <0.001 | 1.86 | 1.60-2.16 | <0.001 |
| Q4 | 2.82 | 2.46-3.23 | <0.001 | 3.60 | 3.10-4.19 | <0.001 |
| p-trend |  |  | <0.001 |  |  | <0.001 |
| Abbreviations: CI, confidence interval; FI, Functional Impairment; OR, odds ratio; WC: waist circumference. ^a^ The associations between WC and Function Impairment among US older adults are presented as ORs (95% CI). Model A did not adjust for any covariates. Model B adjusted for age, sex, race, education level, marital status, alcohol use, smoking—cigarette use, and hypertension, diabetes, coronary heart disease. | | | | | | |
|
|


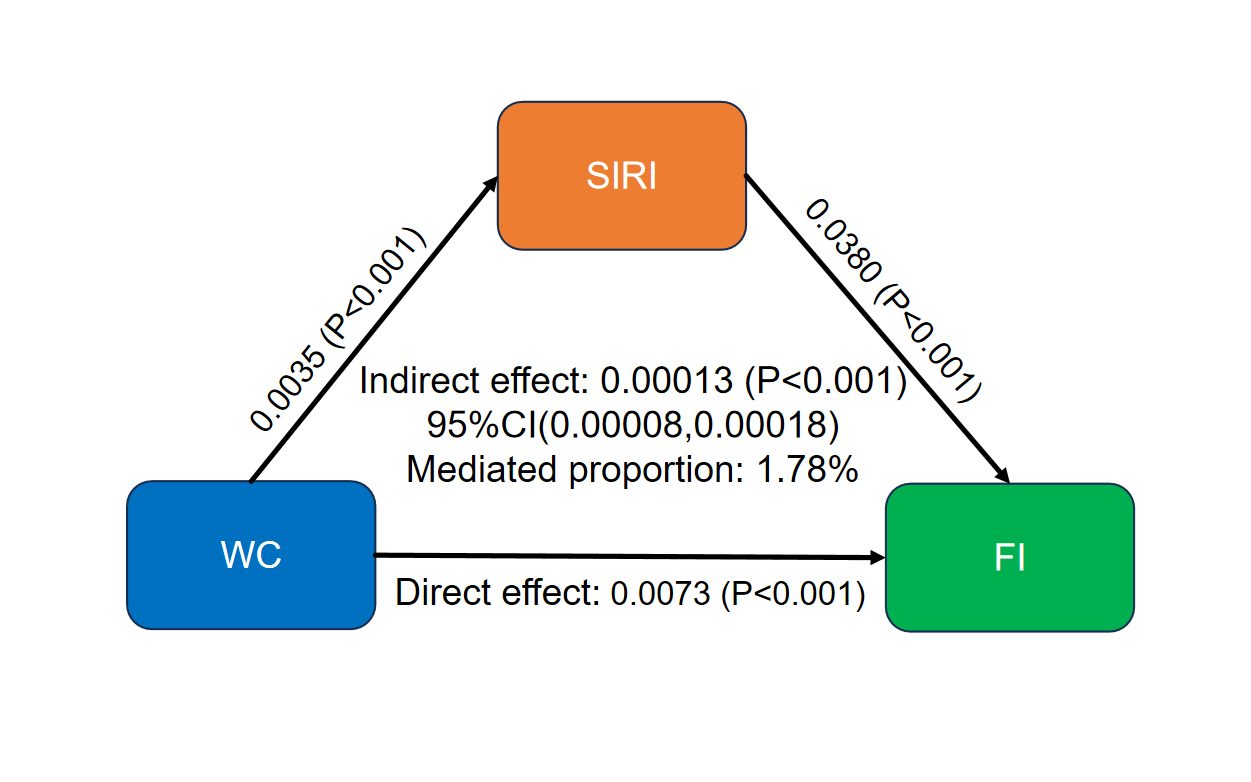


Supplementary Figure 6. Analysis of mediating role of SIRI in the relationship between WC and FI.

Abbreviations: FI, Functional Impairment; SIRI, Systemic Inflammation Response Index; WC, waist circumference.
